# 

**Published:** 1902-01

**Authors:** 


### LIST OF CONTRIBUTORS TO VOLUME XXIII.

Allan, Dr. Charles F.
Allan, Dr. George S.
Anderson, Martha, M.D.
Andrews, R. R., A.M., D.D.S.
Arrington, B. F., D.D.S.
Baker, Lawrence W., D.M.D.
Bogue, E. A., M.D.
Bradley, Frederick, D.M.D.
Briggs, Edward C., D.M.D.
Brown, George V. I., A.B., D.D.S., M.D., C.M.
Carroll, N. G.
Chittenden, Charles C., D.D.S.
Crockett, E. A., M.D.
Curtis, G. Lenox, M.D.
Davenport, S. E., M.D.S., D.D.S.
De Lisle, Dr. Justin.
Eames, George F., M.D., D.D.S.
Engs, John S., D.D.S.
Fillebrown, Dr. Thomas.
Flanagan, Dr. A. J.
Genese, Dr. D.
Gordon, George Byron.
Grant, Harry L., D.M.D.
Howard, W. R., D.D.S.
Hopkins, Samuel A., M.D., D.D.S.
Howe, J. Morgan, M.D.S., M.D.
Inglis, Otto E., D.D.S.
Jack, Louis, D.D.S.
Jackson, Harvey N., D.D.S.
Jenkins, N. S., D.D.S.
Kelley, H. A., D.M.D.
Kirk, Edward C., D.D.S.
Knight, Dr. William.
Latham, V. A., M.D., D.D.S., F.R.M.S.
Lett, Isidore, D.D.S.
Lewis, F. Park, D.D.S.
Linton, Dr. Charles C.
Lloyd-Williams, E., M.R.C.S., L.R.C.P., L.D.S.
McCullough, Dr. P. B.
Macleod, Alexander, D.D.S.
Macvane, S. M., Ph.D.
Noyes, Frederick B., B.A., D.D.S.
Palmer, Dr. S. B.
Parmele, George L., M.D., D.M.D.
Peck, A. H., M.D., D.D.S.
Power, James Edward, D.M.D.
Rollins, William.
Smith, Dr. F. Milton.
Smith, Eugene H., D.M.D.
Spencer, Henry C., D.M.D.
Stoddard, Dr. A. H.
Talbot, Eugene S., M.D., D.D.S.
Trueman, William H., D.D.S.
Wedelstaedt, E. K.
Whitlock, Dr. W. M.
Wright, C. M., D.D.S.

